# What Is New and What Is Next for SAPHO Syndrome Management: A Narrative Review

**DOI:** 10.3390/jcm14041366

**Published:** 2025-02-18

**Authors:** Mario Ferraioli, Juela Levani, Riccardo De Luca, Caterina Matucci-Cerinic, Marco Gattorno, Serena Guiducci, Silvia Bellando Randone, Maria Sole Chimenti

**Affiliations:** 1 Rheumatology, Immunology and Clinical Allergology Unit, Department of Medicina dei Sistemi, University of Rome Tor Vergata, 00133 Rome, Italy; maria.sole.chimenti@uniroma2.it; 2 Division of Rheumatology, Department of Experimental and Clinical Medicine, University of Florence, AOU Careggi, 50121 Florence, Italy; juela.levani@unifi.it (J.L.); riccardodeluca310795@gmail.com (R.D.L.); serena.guiducci@unifi.it (S.G.); silvia.bellandorandone@unifi.it (S.B.R.); 3 Rheumatology and Autoinflammatory Diseases, IRCCS Istituto Giannina Gaslini; DINOGMI, University of Genoa, 16126 Genoa, Italy; caterinamatuccicerinic@gaslini.org (C.M.-C.); marcogattorno@gaslini.org (M.G.)

**Keywords:** SAPHO, SAPHO syndrome, spondyloarthritis, therapy, biologics

## Abstract

Synovitis–acne–pustulosis–hyperostosis–osteitis (SAPHO) syndrome is a rare disease characterized by a sterile inflammatory osteitis and/or arthritis associated with a wide range of dermatological manifestations, such as acne, palmoplantar pustulosis, and psoriasis. This review, providing up-to-date knowledge on this disease, aims at informing researchers and clinicians to help them program future studies in order to improve patients’ care. Due to the vast clinical heterogeneity that characterizes this disease, SAPHO syndrome has received various names; among these, chronic recurrent multifocal osteomyelitis represents the most used one. The various nomenclatures in use also reflect different approaches to its management. Indeed, considering the world-wide distribution and the vast onset age (from children to late adulthood), in addition to the multiform clinical presentation, its diagnosis and treatment are often challenging for clinicians. In this review, we provide valuable insights on SAPHO syndrome, delving into its many aspects: epidemiology, pathogenesis, clinical presentation, diagnosis, and classification. Most importantly, this paper addresses the continuously changing treatment panorama of this disease, from established drugs to newly introduced ones. Furthermore, a peculiar focus regards nonpharmacologic approaches, including traditional Chinese medicine, the apheresis technique, and surgery. Similarly, this review also discusses patients’ lifestyle, including quality of life. To improve SAPHO syndrome’s management, different knowledge gaps should be filled, such as its current epidemiology and pathogenesis. In turn, perfected knowledge in these fields could also advance research in therapy.

## 1. Introduction

The clinical association of synovitis–acne–pustulosis–hyperostosis–osteitis describes a heterogeneous disease known as SAPHO syndrome, characterized by osteoarticular inflammatory lesions associated to a wide variety of dermatological conditions, ranging from acne, psoriasis, and palmoplantar pustulosis to hidradenitis suppurativa, pyoderma gangrenosum, and Sweet syndrome [1].

A first report of peripheral arthritis–acne association dates back to 1961 [2] but, only in 1987, Chamot et al. together with Benhamou were able to describe a unitary syndrome associating rheumatological and dermatological conditions [3,4]. Several nomenclatures have been applied to this syndrome, reflecting the heterogeneity of its rheumatologic and dermatologic manifestations (Table 1). Among the various known terms, chronic recurrent multifocal osteomyelitis (CRMO) represents the most used. CRMO, also known as chronic nonbacterial osteomyelitis (CNO), is an autoinflammatory disease that primarily affects children, characterized by sterile and recurrent osteomyelitis attacks [5], typically involving meta-epiphysis of the long bones, together with the axial skeleton and the anterior chest wall [6]. In children, CRMO is diagnosed based on the criteria proposed by Jansson et al. [7]. However, there is an ongoing debate about whether SAPHO syndrome and CNO are distinct conditions or simply represent different manifestations of the same disease depending on the age at onset [6]. In fact, according to Kahn’s SAPHO diagnostic criteria, the presence of CRMO in children is sufficient for diagnosing SAPHO [8]. Additionally, CRMO can be associated with the same skin manifestations observed in SAPHO syndrome, and association with inflammatory bowel diseases (IBD) has been reported. Also, adult cases of CRMO without skin manifestations have been described [5].

The proliferation of acronyms associated with CRMO/SAPHO, as reported in Table 1, and the fact that these diseases present some similarities with other polygenic autoinflammatory conditions characterized by sterile osteitis and skin manifestations reflect the absence of a comprehensive and uniform classification system, highlighting the need for a renaming of this group of conditions [9]. Similarly, SAPHO syndrome also shares similarities with spondyloarthritis (SpA). For instance, in both diseases, patients may present with peripheral arthritis and enthesitis, involvement of the sacroiliac joint and spine (clinically and radiographically), as well as the presence of psoriasis and IBD [10]. On the other hand, genetic reports on SAPHO syndrome did not show an increase in HLA-B27 frequency, unlike in SpA [1].

By providing the most up-to-date knowledge on SAPHO syndrome, the present review will inform researchers and clinicians involved with this disease. Building on this, this paper aims at helping them to program future studies in order to fill current knowledge gaps and improve patients’ care.

## 2. Literature Review

### 2.1. Methodology

A nonsystematic (PRISMA protocol not followed) narrative literature review was performed on PubMed, including references coming from published papers, congress abstracts, books, and book chapters. The following terms were used as keywords: SAPHO, SAPHO syndrome, synovitis–acne–pustulosis–hyperostosis–osteitis, chronic recurrent multifocal osteomyelitis, therapy, biologic, DMARD, methotrexate, azathioprine, sulfasalazine, cyclosporine, antibiotics, bisphosphonates, phytochemicals, infliximab, adalimumab, etanercept, golimumab, certolizumab, anti-TNFα, anakinra, canakinumab, anti-IL-1, rilonacept, risankizumab, tofacitinib, baricitinib, upadacitinib, anakinra, janus kinase inhibitors, ustekinumab, brodalumab, bimekizumab, ixekizumab, rituximab, abatacept, tocilizumab, anti-IL-6, anti-IL-17, anti-IL-23, anti-IL-12, and anti-TNFα. Then, after discarding non-English references, full texts were screened and the most relevant papers selected.

### 2.2. Epidemiology

SAPHO syndrome is considered as a rare disease, with a reported incidence of 1 in 10,000 in Caucasians [11] and a worldwide distribution, encompassing Europe (especially its northern territories), USA, Latin America, and Asia (Japan and China in particular). To note, the actual incidence is hard to determine as it is often misdiagnosed or misclassified. Disease onset is considered between the third and fifth decade of life [12]. In children, CRMO has a reported prevalence of 0.4 per 100,000, with predilection for females [13]. Hayem et al. [11] found a higher female prevalence (7:5) when the disease onset is before 30 years of age, even if following reports have highlighted a different prevalence in terms of sex and age of onset depending on the skin manifestations; severe acne is more frequent in men, whereas palmoplantar pustulosis and psoriasis are more common in females [14]. The same distribution has been highlighted also in children [9].

### 2.3. Pathogenesis

**Figure 1:**
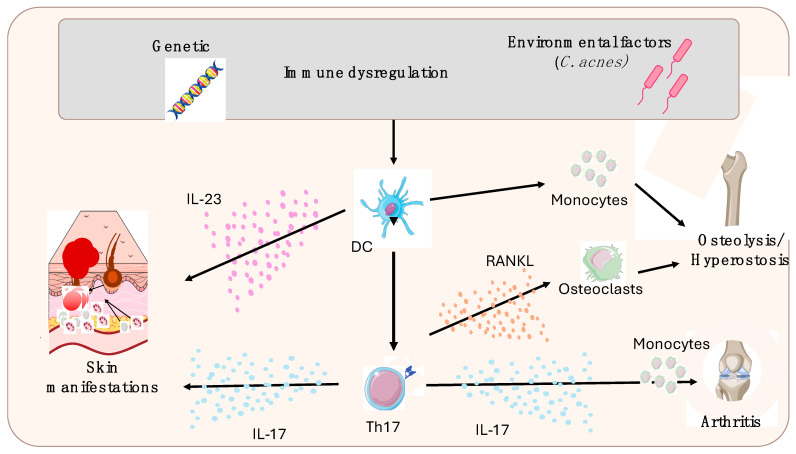
Pathogenesis of SAPHO syndrome. A complex interaction between genetic predisposition, environmental factors, and immune dysregulation leads to the loss of tolerance that, in turn, produces the activation of the immune system. The T helper 17 cells (Th-17) appear to be pivotal in this process. In fact, IL-17 production leads to the typical skin disorders related to psoriasis (together with IL-23) and acne. On the other hand, Th-17 mediates the osteoarticular manifestations, such as peripheral arthritis, and—by activating osteoclasts—bone damage (osteolysis and hyperostosis). Figure legend: IL: interleukin; DC: dendritic cell; Th: T helper cell; RANKL: receptor activator of nuclear factor kappa-Β ligand.

SAPHO syndrome pathogenesis is still uncertain, and its development is probably due to an intricate interaction between immune dysregulation, genetic susceptibility, and environmental factors (Figure 1). At the same time, it is still discussed if SAPHO should be classified among immune-mediated or auto-inflammatory diseases. Even though the frequent involvement of the axial skeleton, enthesitis, psoriasis, and IBDs may point SAPHO syndrome’s nosology towards the SpA group, some common features with other auto-inflammatory conditions make the latter model more likely. In fact, reduced natural killer (NK) cells, elevated levels of different proinflammatory interleukins, and a positive response to anti-IL-1 treatments, as well as a neutrophil hyperactivity, have been highlighted in in vivo studies [15,16,17]. Additionally, SAPHO syndrome shares some clinical similarities with several monogenic auto-inflammatory syndromes, such as Majeed syndrome, deficiency of IL-1 receptor antagonist (DIRA), and pyogenic arthritis, pyoderma gangrenosum, acne (PAPA) syndrome [18,19], and with a number of multifactorial autoinflammatory conditions characterized by the presence of sterile osteitis associated with skin manifestations that have been included in the so-called “PAPA spectrum disorders”, such as pyogenic arthritis, pyoderma gangrenosum, acne, suppurative hidradenitis (PAPASH), psoriatic arthritis, pyoderma gangrenosum, acne, suppurative hidradenitis (PsAPASH), and pyoderma gangrenosum, acne vulgaris, hidradenitis suppurativa, and ankylosing spondylitis (PASS) syndromes [20,21].

Data from animal models supported a possible auto-inflammatory origin of SAPHO, including the IL-1 receptor antagonist deficient mice (that show inflammatory bone and skin manifestations related to T helper (Th)-17 cells) [22] and the PSTPIP2 knock-out mouse model (which presents synovitis, hyperostosis, osteitis, and multifocal osteomyelitis) [23,24]. In the latter model, mice also showed elevated blood levels of IL-1. This could possibly be explained with results from both in vitro and mouse models that showed the intrinsic capacity of *Prevotella* sp. (especially from the gut) to induce IL-1β and IL-23 production, inducing a Th17 response [25]. However, the promising findings of PSTPIP2 mutated mice have not been confirmed in human cohorts [26].

Genetics of SAPHO syndrome have been variously studied and a familiar predisposition proposed, even though no specific gene has definitively been linked to this disease [27], unlike in SpA [28]. Human leukocyte antigens (HLA) genes have been taken into account, like HLA-A26, -B27, -B39, and -B61, but their association was not confirmed [1,29]. Other non-HLA genes have also been implied, including LPIN2, PSTPIP2, and NOD2, but no specific or pathogenic association emerged [26]. Also, the possible role of the FBLIM1 gene has not been confirmed in other studies [30,31]. Conversely, a transcriptome analysis found overactive neutrophil recruitment genes, including ITGB2, CCRL2, and CD151 [15]. Additionally, the evidence of abnormal cytokine levels (IL-1, IL-8, IL-17, IL-18, and tumor necrosis factor (TNF)-α) in SAPHO patients highlighted a possible involvement of the Th-17 pathway [16,32]. To this regard, it has been proposed that a reduction in NK cells might cause an imbalance between Th17 and regulatory T cells that, in turn, overstimulates the innate immune system, leading to an altered capacity to control pathogens (such as *Cutibacterium acnes*), resulting in chronic inflammation [17].

Lastly, different infectious pathogens have been associated with SAPHO syndrome, some isolated from bony lesions. The most commonly reported is *Cutibacterium* (previously known as *Propionibacterium*) *acnes*, followed by *Staphylococcus aureus*, *Haemophilus parainfluenzae*, and *Actinomyces* [33]. Among these, *C. acnes*, a Gram-positive, anaerobic commensal bacterium found in some bone cultures, may be a suitable connection between the two main SAPHO syndrome features: bone and skin lesions [34,35]. In 1987, Trimble et al. demonstrated that intra-articular injection of *C. acnes* in rats led to an erosive arthritis associated to bone erosions [35]. It has been theorized that *C. acnes* may be able to escape immune control and transform into a pathogen due to a genetic or molecular defect that impairs its elimination [32,34]. This process may involve a reduction in NK cells, paired with a downregulation of the Fork-head Box O1 (FoxO1) protein, which is responsible for clearing the bacterium [32,34]. Complement-mediated immune activation by *C. acnes*, leads to the release of proinflammatory cytokines, like IL-1, -8, -17A, and TNFα, that, in turn, mediate skin and bone pathological involvement [17,32].

Notably, *C. acnes* is also implied in acne, a pivotal component of SAPHO and a condition based not only on inflammation but also on oxidative stress. Indeed, acne skin tissues show a pro-oxidative environment with reduced levels of glutathione peroxidase (GTPx), superoxide dismutase, and glutathione [36,37]. Furthermore, catecholamines have also been implied in acne pathogenesis, as well as in *C. acnes*. On one hand, catecholamines can stimulate sebum production in sebaceous glands and, on the other hand, it has been shown how they favor *C. acnes* in vitro formation of biofilm [38]. Building on this evidence, Bungau et al. have recently evaluated a group of acne patients, describing a significant negative association between severity of acne and urinary GTPx, as well as a positive association with noradrenaline urinary levels [39].

### 2.4. Clinical Presentation

SAPHO syndrome may present with a plethora of clinical manifestations. Bone and joint involvement represent the typical features of the disease that may be accompanied by systemic symptoms and involvement of other organs. Table 2 illustrates the most prominent rheumatologic and dermatologic characteristics and their frequency.

#### 2.4.1. Rheumatological Findings

Osteoarticular features are characterized by pain, swelling, and tenderness of the involved areas that may be chronic or episodic-relapsing, focal, or multifocal [40,41,42,43]. Pathologic lesions include (1) inflammatory arthritis (synovitis), usually nonerosive, (2) osteitis (a cortical and/or medullary cavity focal inflammation), (3) hyperostosis (a bony overgrowth due to endosteal and/or periosteal proliferation), and (4) axial SpA-like changes affecting the sacroiliac joints, spine, and enthesitis [29,40,41,43,44,45,46,47,48,49,50,51]. They can all be concurrent and be present at the same time. The most commonly affected regions include the anterior chest wall, present in up to 90% of cases and considered characteristic of SAPHO syndrome, sacroiliac joints, as well as the spine (up to 90% of subjects) and peripheral joints (almost 30%) such as hip, knee, and ankle [1,52] (Table 2). Different authors have also reported orofacial manifestations, involving diffuse and sclerosing osteomyelitis of the mandible or the maxillary bone [53] and isolated hyperostosis of the frontal [54] and mandibular bone [55]. Associated symptoms may include trismus, soft tissue swelling, as well as dysphagia, paresthesia, and deformation [53].

#### 2.4.2. Dermatological Findings

Skin lesions have a wide range of severity and are composed of different acneiform and neutrophilic dermatoses. Hayem et al. reported that 68% of patients had skin involvement at diagnosis or before [40]. The two most common manifestations are palmoplantar pustulosis (PPP), present in up to 60% of cases, and severe acne, in about 20% of patients [56]. PPP is characterized by chronic, recurrent sterile pustules involving palms and plants [57]. Acne can present as nodulocystic acne or acne fulminans; it is generally severe and difficult to treat, leading to disfiguring scars. Also, plaque psoriasis can be present, isolated, or associated to PPP in 5–20% of patients (Table 2). Moreover, other neutrophilic dermatoses have been described in SAPHO, such as pyoderma gangrenosum or hidradenitis suppurativa (both of which can be isolated or associated to acne) and Sweet syndrome [1].

Notably, skin and osteoarticular manifestations have a variegated temporal relationship. Indeed, dermatological findings can be absent, precede, be concomitant, or even follow rheumatological ones. However, the majority of patients (70%) develop both manifestations within two years of disease onset [41,42,58].

#### 2.4.3. Other Clinical Features

Systemic symptoms, such as fatigue and fever, can be present [1].

An association with IBD—which are also associated to neutrophilic dermatoses and SpA—has been known since SAPHO’s description [40] and is also part of the modified 2003 Kahn’s criteria [8].

Moreover, the presence of venous thrombosis, especially of the subclavian vein has been largely reported, presumably resulting from an hyperostotic compression [59,60].

Lastly, few reports have described anecdotical associations with hypertrophic pachymeningitis [61], uveitis [62], AA amyloidosis [63], and pleuro-pulmonary abnormalities [64,65].

#### 2.4.4. Laboratory Findings

Laboratory findings are usually nonspecific and no serum markers have been discovered yet. Inflammatory markers might be elevated, with erythrocyte sedimentation rate (ESR) and C-reactive protein (CRP) reported to be elevated in about 10–50% of patients [1,40]. HLA-B27 positivity varies greatly, being reported in about 4–13% of patients [40,66]. Since it does not differ significantly from the general population, it is not considered a disease marker.

#### 2.4.5. Radiological Findings

Radiological findings mainly describe the osteoarticular changes. Plain radiography of peripheral bones may show hyperostotic and/or erosive changes, sclerotic lesions, osteolysis, periosteal reaction/osteopenia, joint space narrowing, and osteoproliferation in-volving entheses [43,52,66]. The same technique is used to evaluate axial involvement, where both osteitis and ankyloses may be seen. Typical signs are vertebral body corner lesions, nonspecific spondylodiscitis, osteo-destructive and osteosclerotic lesions, paravertebral ossification, and sacroiliitis [47,52,67]. Bone scintigraphy has many advantages, as it can assess multiple involved sites, as well as rule out malignancy and infection. The ‘‘bull’s head’’ sign, seen with this technique, describes a symmetric uptake in the sternoclavicular region; it is characteristic but not highly sensitive [68].

Magnetic resonance imaging (MRI) has been suggested as a gold standard diagnostic modality for its ability to detect osteitis (bone marrow oedema) as well as structural lesions, like erosions and ankyloses [68]. Fritz et al. found that, at early stages, conventional radiography only shows 16% of the lesions seen on MRI [69]. Moreover, Zhang et al. illustrated some distinct SAPHO syndrome MRI features compared to SpA [70]. The former, in fact, had lower incidence of both sacroiliitis and erosions, as well as a higher incidence of anterior chest wall involvement.

Fluorodeoxyglucose Positron Emission Tomography has demonstrated great use-fulness in differentiating SAPHO lesions from tumoral metastatic lesions [71].

Lastly, ultrasonography with color power-Doppler has proven to be efficacious in detecting active synovitis in peripheral and sternoclavicular joints as well as discriminating SAPHO syndrome patients from controls [72].

#### 2.4.6. Comorbidities

Unfortunately, few data are available on the matter. Przepiera-Będzak et al. reported hypertension (25%) as the most common comorbidity, followed by hypothyroidism and diabetes (9.6% each) [73]. The same authors also suggested an increased risk of cardio-vascular disease in SAPHO patients since they found a higher prevalence of metabolic syndrome than controls [69]. Few studies demonstrated also an association of SAPHO patients and their relatives with autoimmune diseases [27,74]. Chen et al. found an in-creased prevalence of osteoporosis in SAPHO patients compared to healthy controls [44]. Moreover, Li et al. highlighted a higher sexual dysfunction and lower values at mental health assessment in female SAPHO patients [75]. Lastly, a recent Chinese study found an 18% prevalence of fibromyalgia, which was also associated to female sex and older age [76].

#### 2.4.7. Diagnosis and Differential Diagnosis

Diagnosis of SAPHO syndrome can be challenging, being mainly based on clinical and radiological presentation, as no specific diagnostic marker has been developed yet. SAPHO syndrome should be suspected when an inflammatory arthritis and/or osteitis (particularly if involving the anterior chest wall, sacroiliac joints, or spine) is associated with psoriasis or a neutrophilic or acneiform dermatosis.

Several subsets of diagnostic criteria (Table 3) have been proposed, but none of them have been validated. They are all based on the association between a bone and/or joint manifestation with a form of SAPHO’s typical skin disease (present or past). The first diagnostic criteria were published by Benhamou et al. in 1988 [3], followed in 1994 by Kahn’s criteria [77], which were subsequently modified by the same author in 2003 [8]. These criteria are helpful in diagnosis, even though they need further validation.

Lastly, a number of differential diagnoses should be kept in mind when evaluating a patient, including:

- Rheumatologic diseases: rheumatoid arthritis (that can be associated to neutrophilic dermatoses) and SpA (including psoriatic arthritis) that may also associate with IBD. In this case, osteoarticular pattern may distinguish SAPHO syndrome.
- Infectious diseases: infectious osteomyelitis or spondylodiscitis.
- Malignancies: osteosarcoma, Ewing’s sarcoma, bone metastasis, Paget’s disease, bone lymphoma, or histiocytosis.
- Monogenic autoinflammatory bone diseases: PAPA syndrome, DIRA syndrome, deficiency of interleukin-36 receptor antagonist (DITRA) syndrome, or Majeed syndrome.

It should be noted that, the infectious and neoplastic hypothesis should be always ruled out, especially when confronting a monostotic lesion, by means of a bone biopsy.

### 2.5. Treatment

SAPHO syndrome’s treatment, considering the large clinical spectrum of the disease and the virtual absence of any randomized clinical trial, remains a challenge. Medications are usually selected based upon the specific manifestations, and treatment is mainly guided by case reports, case series, or expert opinion. Many drugs have been used, including steroids, antibiotics, nonsteroidal anti-inflammatory drugs (NSAIDs), bisphosphonates, conventional and biological disease-modifying antirheumatic drugs (cDMARDs and bDMARDs), as well as small molecules, among others.

#### 2.5.1. NSAIDS, Steroids, Antibiotics, Bisphosphonates, and cDMARDS

NSAIDs represent the primary initial treatment for osteoarticular manifestations in the great majority of cases [14,40,78]. There is no particular drug preferred over another. Their efficacy in reducing osteoarticular symptoms is transient and, in most cases, the addition of another drug is needed.

Colchicine has been used due to its immune-modulatory effect, being able to reduce the dysregulated chemotactic activity highlighted in SAPHO syndrome [79]. It is complex to determine its real efficacy since, like NSAIDs, it is often used in combination with other drugs; however, it was reported effective in some cases of pustulotic arthro-osteitis [80,81,82].

Corticosteroids are usually administered for short periods of time, proving to be efficacious especially in patients with the acne phenotype. For peripheral arthritis, intra-articular injections can be of help.

Antibiotic therapy for SAPHO syndrome represents another possible therapeutical approach, considering that C. acne may act as a trigger. Tetracyclines (doxycycline and minocycline) and azytromicine are typical first-line agents (as in acne vulgaris), usually used for prolonged times. However, only a small proportion of patients has a clinical response to treatment [29,35,40,43,82], and the effect is often partial and lost after treatment withdrawal.

Bisphosphonates are commonly used and they act by inhibiting bone resorption and turnover. Their cumulative efficacy is difficult to calculate, since they are often used in association with other drugs, and ranges around 50% [46]. Pamidronate is the most frequently used. Different reports outlined its efficacy, especially on spinal involvement. Interestingly, it is the only nonbiologic drug in the SAPHO syndrome treatment landscape for which a clinical trial is available [83]. The study included 14 patients and results concluded that pamidronate reduced bone marrow oedema and improved pain and disability. Other reports describe satisfying effects after pamidronate administration both in adults and children [29,84,85,86,87]. Recently, Sakellariou et al. reported a complete resolution after three injections of denosumab in a patient with anterior chest wall and axial skeleton involvement [88].

**Table 2:** Prominent rheumatologic and dermatologic characteristics of SAPHO syndrome.

	Patients, n	Anterior Chest Wall, n (%)	Spine, n (%)	Sacroiliac, n (%)	Long Bone, n (%)	Mandible, n (%)	Peripheral Arthritis, n (%)	Palmoplantar Pustulosis, n (%)	Psoriasis Vulgaris, n (%)	Acne, n (%)
Hayem et al. (1999) [40]	120	63 (52.5)	33 (27.5)	40 (33.3)	6 (5)	11 (9.16)	36 (30)	55 (45.83)	10 (8.33)	18 (15)
Sallés et al. (2011) [49]	52	-	1 (1.92)	27 (51.9)	-	1 (1.92)	17 (32.7)	17 (32.6)	11 (21.1)	13 (25)
Colina et al. (2009) [86]	71	50 (70.4)	24 (33.8)	13 (18.3)	0	3 (4.22)	28 (39.4)	20 (28.1)	3 (4.22)	9 (12.6)
Aljuhani et al. (2015) [29]	41	28 (68.2)	16 (39.0)	12 (29.2)	10 (24.2)	2 (4.87)	24 (58.5)	19 (46.3)	3 (7.31)	2 (4.8)
Huang et al. (2021) [45]	24	15 (62.5)	14 (58.3)	2 (8.3)	17 (70.8)	3 (12.5)	-	10 (41.6)	-	7 (29.1)
Przepiera-Będzak et al. (2021) [78]	46	45 (97.8)	-	-	-	-	-	42 (91.3)	-	3 (6.52)
Li et al. (2022) [47]	376	354 (94.1)	233 (61.9)	()	()	()	232 (61.7)	294 (78.2)	78 (20.74)	50 (13.3)
Yap et al. (2021) [50]	21	16 (76.1)	-	13 (61.9)	-	10 (47.61)	-	4 (19.0)	-	2 (9.52)
Cao et al. (2019) [42]	335	301/340 (88.5)	140/340 (41.1)	110/340 (32.3)	56/340 (16.4)	120/340 (35.3)	-	308/335 (91.9)	53/335 (15.82)	48/335 (14.3)
Li et al. (2016) [41]	164	128/157 (81.5)	71/157 (45.2)	46/157 (29.2)	15/157 (9.5)	54/157 (34.4)	-	108 (65.8)	24 (14.63)	11 (6.7)
Total	1331	1068	582	270	46	91	521	947	182	174
Mean		106.8	64.6	33.7	11.5	13	65.1	86.1	26	15.8
(95% CI)		(114.08–99.52)	(70.81–58.51)	(37.81–29.69)	(12.84–10.16)	(17.04–8.96)	(71.63–58.61)	(79.03–93.15)	(21.82–30.18)	(13.27–18.36)

**Table 3:** SAPHO diagnostic criteria.

Benhamou et al. 1988 Criteria [3]	Kahn et al. 1994 Criteria [77]	Kahn et al. 2003 Criteria [8]
Inclusion features:	Any of the three presentations is sufficient for diagnosis	Inclusion:
Osteo-articular manifestations of acne conglobata, acne fulminans, or hidradenitis suppurativa	1. Chronic recurrent multifocal osteomyelitis	Bone ± joint involvement associated with PPP and PV
Osteo-articular manifestations of PPP	○ Usually sterile	Bone ± joint involvement associated with severe acnes
Hyperostosis (of the anterior chest wall, limbs, or spine) with or without dermatosis	○ Spine may be involved	Isolated sterile hyperostosis/osteitis (adults)
CRMO involving the axial or peripheral skeleton with or without dermatosis	○ With or without skin condition	Chronic recurrent multifocal osteomyelitis (children)
Sometimes reported:	2. Acute, subacute, or chronic arthritis associated with any of the following:	Bone ± joint involvement associated with chronic bowel diseases
- Possible association with psoriasis vulgaris	○ Palmoplantar pustulosis	
- Possible association with an inflammatory enterocolopathy	○ Pustular psoriasis	
- Features of ankylosing spondylitis	○ Severe acne	
- Presence of low-virulence bacterial infections	3. Any sterile osteitis * associated with any of the following:	
Exclusion features:	○ Palmoplantar pustulosis	Exclusion:
Septic osteomyelitis	○ Pustular psoriasis	Infectious osteitis *
Infectious chest wall arthritis	○ Psoriasis vulgaris	Tumoral conditions of bone
Infections PPP	○ Severe acne	Noninflammatory condensing bone lesions
Palmo-plantar keratodermia		
DISH except for fortuitous association		
Osteoarticular manifestations of retinoid therapy		
The presence of 1 of the 4 inclusion features is sufficient for a patient to be included in the SAPHO syndrome.		

Table’s Legend: PPP: palmoplantar pustulosis; CRMO: chronic recurrent multifocal osteomyelitis; DISH: diffuse idiopathic skeletal hyperostosis; PV: psoriasis vulgaris. * except for *Cutibacterium acnes*.

csDMARDs (methotrexate, sulfasalazine, cyclosporine, and leflunomide) are another class of drugs highly used in SAPHO syndrome. Methotrexate is the most frequently administrated, especially for its efficacy on peripheral arthritis and dermatological manifestations [29,40,51,55,67,78,89,90,91,92]. Few data are reported on cyclosporine, which has been mainly used for cutaneous involvement [92]. Sulfasalazine has also been used, often as an additional therapy [9,29,40,44,78]. Leflunomide represents the less frequently reported cDMARD in SAPHO syndrome and its effects are poorly discussed [92]. Interestingly, a case report described its efficacy on nail involvement [93].

Lastly, Adamo et al. [94] reported satisfactory results using a pan-cytokine approach with Apremilast, a PDE-4 inhibitor, used to treat psoriasis and PsA, which was efficacious in treating one patient with PPP and sternoclavicular involvement. Conversely, other papers reported unsatisfactory effects on both skin and joints [95].

#### 2.5.2. Biologics

bDMARDs and targeted synthetic DMARDs (tsDMARDs) have been variously used in SAPHO syndrome. Even though their use is mostly limited to refractory cases, their efficacy has been largely highlighted. Among the different drug types, anti-TNFα agents (Adalimumab, Etanercept, Infliximab, Certolizumab, and Golimumab) are the most frequently mentioned in the literature, followed by anti-IL-17 agents (Secukinumab, Brodalumab, and Bimekizumab), anti-IL-12/23 (Ustekinumab), anti-IL19p23 (Risankizumab), antiIL-1 (Anakinra), and anti-IL-6: Tocilizumab). Recently, small molecules like the inhibitors of the Janus Kinases (JAK) signaling pathway (Tofacitinib, Baricitinib, and Upadacitinib) have also been introduced. To note, most of the reports about b- and tsDMARDs are case reports describing one patient. In Supplementary Table S1, an overview of all the patients treated with biologics is reported.

Overall, while the reported biologics have a good efficacy in osteoarticular involvement, the skin manifestations, especially acne, hidradenitis suppurativa, and pyoderma gangrenosum, appear to be particularly refractory to treatments. The largest reports in the literature regard anti-TNFα agents (Infliximab, Adalimumab, and Etanercept), while very few cases cover Certolizumab’s and Golimumab’s use. In the different case series, Adalimumab (ADA) was the most used drug [9,45,96,97,98,99,100,101,102,103,104,105,106,107,108,109,110,111,112,113], followed by Infliximab (IFX) [96,101,114,115,116,117,118,119,120,121,122,123,124,125,126,127,128] and Etanercept [9,45,96,105,112,126,127,128,129,130,131,132,133,134,135]. Both ADA and IFX were efficacious on the osteoarticular symptoms in almost all the treated patients (Supplementary Table S1: 32/35 ADA, 27/29 IFX), with a good response also on skin manifestations (14/19 IFX, 27/31 ADA). Specifically, IFX was efficacious on 6 of the 7 patients with acne/hidradenitis suppurativa (HS) and in 7 of the 10 treated PPP, while ADA was effective in inducing a remission in 14/17 PPP (with 2 partial responses) and in 10 acne-HS out of 14 treated patients.

On the other hand, some paradoxical cutaneous effects after the introduction of a biologic therapy have been reported, especially regarding psoriasis and hidradenitis suppurativa [136]. Interestingly, Gimeno-Castillo et al. illustrated the paradoxical development of SAPHO syndrome (positive bone scintigraphy) in a young female patient with skin lesions compatible with PPP 6 weeks after Etanercept introduction [137].

Finally, a multicenter randomized double-blind clinical phase 2/3 is currently ongoing to evaluate Etanercept’s efficacy and safety in the treatment of SAPHO syndrome (NCT06011889).

Following TNFα inhibitors, anti-IL agents have also been used and particularly those derived from SpA treatment. Anti-IL17 are the most described with a generally good efficacy profile. Interestingly, the two case series that describe Secukinumab—the most reported drug—illustrate contradictory results [9,109,110,111,138,139,140,141,142,143]. In fact, while Wang et al. [138] described a satisfactory efficacy on both joints and skin symptoms in all of their four patients, no particular efficacy on the osteoarticular manifestations, and only a partial efficacy on skin was reported by Wendling et al. in a series of three patients [139]. The other single cases reported instead a good efficacy on bone skin and bone manifestations (overall 11/15 skin resolution and 11/16 bone remission). Fewer data are available regarding the other anti-IL-17 drugs. Few cases report an efficacy with Ixekizumab [144], Bimekizumab [145], as well as with Brodalumab [146,147].

Concerning Ustekinumab—an anti-IL-12/23p40 monoclonal antibody, few reports have been presented [9,112,139]. A case series illustrated an improvement in both PPP and peripheral arthritis only in one out of three treated patients, and the development of paradoxical psoriasis was reported in one of the subjects [99].

Risankizumab, an anti- IL-23p19, has also been described as a potential treatment option [109,148] with satisfactory results on all the components, including quality of life (QoL) [109].

Eventually, Anakinra (anti-IL-1) was tried in a few patients. Wendling et al. observed a clinical response in five out of six patients, mainly on the articular manifestations [149].

Lastly, JAK inhibitors have been recently used to treat SAPHO syndrome [9,150,151,152,153,154,155]. In particular, Tofacitinib, a JAK1 and -3 inhibitor, has shown an efficacy on both skin and joint involvement. Two different pilot studies have reported promising results: Li et al. treated 12 patients; 9 had a bone response and 7/8 experienced an improvement in skin manifestations (6 PPP and 1 acne). Additionally, all of them reported a significant improvement in pain [151]. Subsequently, another study found a significant improvement in both PPP and nail involvement in 13 SAPHO patients, without, however, a clear efficacy on the osteoarticular manifestations (reduction in pain and no MRI improvement reported) [152]. Interestingly, Ru et al. [153] reported treating a patient with coexisting Takayasu syndrome, describing reduction in both skin and osteoarticular symptoms, as well as an improvement in carotid artery thickness.

As for Baricitinib, while a first report only obtained osteoarticular improvements on a single patient [156], Liu et al. reported an overall improvement in five patients treated with this JAK1-2 inhibitor [157]. Lastly, one patient had a good response on both skin and osteoarticular manifestations with the JAK-1 inhibitor Upadacitinib [158].

#### 2.5.3. Beyond Biologics

Several reports have been published about the use of *Tripterygium wilfordii hook f*, a traditional Chinese medicine herb already used to treat some chronic inflammatory diseases, like rheumatoid arthritis. Since their first case report in 2017, this vine has gained increasing interest in the treatment of SAPHO syndrome [159,160]. Furthermore, some phytochemicals have been tested in psoriasis due to their immune-modulatory and antioxidant characteristics that may influence cellular responses in this condition [161,162]. Among these, polyphenols (like delphinidin, quercetin, and curcumin) showed anti-psoriatic activities in different research settings. In a mouse model of psoriasis, topical treatment with delphinidin significantly reduced inflammatory cell infiltration and lowered the levels of proinflammatory cytokines [162]. Similarly, in another mouse model, topical quercetin improved psoriasis symptoms, reducing oxidative stress markers and downregulating the NF-κB pathway [162]. Furthermore, a rising amount of evidence has highlighted the beneficial role of curcumin in psoriasis from both mouse models and in vivo studies [162]. Particularly, a recent meta-analysis reported preclinical data showing curcumin capacities to inhibit cell proliferation and cycle, as well as production of IL17, TNF, and IL22 [163]. Additionally, authors concluded that both curcumin monotherapy and in combination are able to reduce psoriasis disease activity [163].

Moreover, a non-drug-based approach has been proposed featuring granulocyte and monocyte adsorption apheresis. This technique has been suggested for refractory cases and applied by Kawakami et al. [164] on 10 patients with pustulotic arthro-osteitis, observing an improvement of the osteoarticular symptoms in five patients and of PPP in two out of five patients.

Additionally, non-systemic topical therapies for the treatment of skin manifestations have also been applied, such as topical retinoids, betamethasone, and calcipotriol ointments, as well as the association of psoralen and ultraviolet light radiation. Notably, nonconclusive data are available regarding any of these, as they are always associated with other treatments.

Also, for the treatment of severe acne, oral isotretinoin is widely used in clinical practice [165], with a good efficacy reported in SAPHO cases, even if always combined with other therapies [166,167,168]. However, isotretinoin is known to trigger acne fulminans in patients with severe acne when initiated at high doses [169], with some cases reporting an exacerbation of the osteoarticular symptoms after high doses of isotretinoin in SAPHO [170,171,172].

Lastly, an interesting correlation has been highlighted with tonsillectomy. In fact, as in PPP, an association of tonsillitis with SAPHO has been suggested [173] and some re-ports have described symptom improvement after surgical treatment [173,174].

### 2.6. Lifestyle

Many traits of SAPHO syndrome may affect patients’ QoL, an aspect of this disease poorly assessed in the literature. Witt et al. explored QoL through the SF-36 questionnaire, highlighting that many patients underwent relevant limitations, mainly in the general health, widespread pain, and vitality dimensions. Moreover, osteoarticular manifestations had the strongest impact on the overall disease burden [175].

Diagnostic delay has also been correlated to poor QoL, since it resulted in chronic and potentially debilitating symptoms [176].

Another study reported that SAPHO patients suffered from a cosmetic disability due to active skin involvement, capable of negatively affecting their interpersonal contacts [78]. The same researchers also highlighted an increased risk of depression in SAPHO patients. Moreover, Li et al. [48] found that SAPHO patients also experienced a decreased work productivity and activity, which also correlated with different disease-related measures.

In this context, physicians’ intervention is essential to maximize health-related QoL as well as preventing disabilities and preserving social functions. Among others, smoking habit should be ceased as it is highly associated with PPP, and many patients experienced improvements in skin symptoms after smoking cessation [79].

## 3. Future Approaches

Many questions still remain unanswered regarding SAPHO syndrome. Among others, updated epidemiology studies are lacking, as well as reliable classification criteria. Both, in fact, would allow better guiding of clinical trials and patients’ management. Similarly, given that current medications seem to be less effective compared to those used for other rheumatologic conditions, it is crucial to focus on understanding the mechanisms of the disease. Specifically, grasping the intricate interplay between skin and bone symptoms, which is the defining feature of the condition, could pave the way for novel therapeutic strategies.

Secondly, updated classification criteria would help navigate SAPHO’s clinical heterogeneity, ultimately improving patients’ care. In fact, improving standardization would profit epidemiologic studies, that are essential to clinical practice and to program clinical trials.

Lastly, since only a few studies have assessed QoL, this item should also be highly prioritized to better understand patients’ needs and to assess the impact of treatments.

In conclusion, SAPHO syndrome is a peculiar disease that poses diagnostic and therapeutic challenges to clinicians. In order to improve its management, a multidisciplinary approach is mandatory as well as a collaboration among researchers.

## Figures and Tables

**Table 1:** Previous and alternative nomenclatures used for SAPHO syndrome.

Acquired Hyperostosis Syndrome
Anterior Thoracic wall Inflammatory syndrome
Recurrent Relapsing Symmetrical Clavicular osteitis
Sterno-Costo-Clavicular Hyperostosis
Inter-Sterno-Costo-Clavicular Ossification
Pustulotic Arthro-Osteitis
Spondyloarthritis Hyperostotica Pustulo-Psoriatica
Chronic Recurrent Multifocal Osteomyelitis (CRMO)/Chronic Nonbacterial Osteitis (CNO)
Chronic Mandibular Osteitis
Bilateral Clavicular Osteomyelitis with Palmar And Plantar Pustulosis
Arthro-Osteitis Associated with Follicular Occlusive Triad

## Data Availability

No new data were created or analyzed in this study. Data sharing is not applicable to this article.

## Supplementary Materials

The following supporting information can be downloaded at: https://www.mdpi.com/article/10.3390/jcm14041366/s1, Supplementary Table S1: Reports on biologic and small-molecule drugs used in SAPHO syndrome.

## Abbreviations

The following abbreviations are used in this manuscript:

SAPHO	Synovitis–Acne–Pustulosis–Hyperostosis–Osteitis
CRMO	Chronic Recurrent Multifocal Osteomyelitis
CNO	Chronic Nonbacterial Osteomyelitis
IBD	Inflammatory bowel diseases
SpA	Spondyloarthritis
NK	Natural Killer
IL	Interleukin
DIRA	Deficiency of IL-1 receptor antagonist
PAPA	Pyogenic Arthritis, Pyoderma gangrenosum, Acne
PAPASH	Pyogenic Arthritis, Pyoderma gangrenosum, Acne, Suppurative Hidradenitis)
PsAPASH	Psoriatic Arthritis, Pyoderma gangrenosum, Acne, Suppurative Hidradenitis
PASS	Pyoderma gangrenosum, Acne vulgaris, hidradenitis Suppurativa, and ankylosing Spondylitis
Th	T helper
HLA	Human leukocyte antigens
TNF	Tumor Necrosis Factor
GTPx	Glutathione peroxidase
PPP	Palmoplantar pustulosis
MRI	Magnetic resonance imaging
DITRA	Deficiency of interleukin-36 receptor antagonist
NSAIDs	non-steroidal anti-inflammatory drugs
DMARDs	Disease Modifying Antirheumatic Drugs
JAK	Janus Kinases
ADA	Adalimumab
IFX	Infliximab
HS	Hidradenitis suppurativa
QoL	Quality of Life

## References

[1] Rukavina I. (2015). SAPHO Syndrome: A Review. J. Child. Orthop..

[2] Windom R.E., Sanford J.P., Ziff M. (1961). Acne Conglobata and Arthritis. Arthritis Rheum..

[3] Benhamou C., Chamot A.M., Kahn M.F. (1988). SAPHO syndrome. Ann. Dermatol. Venereol..

[4] Chamot A.M., Benhamou C.L., Kahn M.F., Beraneck L., Kaplan G., Prost A. (1987). Acne-pustulosis-hyperostosis-osteitis syndrome. Results of a national survey. 85 cases. Rev. Rhum. Mal. Osteoartic..

[5] Hedrich C.M., Morbach H., Reiser C., Girschick H.J. (2020). New Insights into Adult and Paediatric Chronic Non-Bacterial Osteomyelitis CNO. Curr. Rheumatol. Rep..

[6] Ferguson P.J., Sandu M. (2012). Current Understanding of the Pathogenesis and Management of Chronic Recurrent Multifocal Osteomyelitis. Curr. Rheumatol. Rep..

[7] Jansson A., Renner E.D., Ramser J., Mayer A., Haban M., Meindl A., Grote V., Diebold J., Jansson V., Schneider K. (2007). Classification of Non-Bacterial Osteitis: Retrospective Study of Clinical, Immunological and Genetic Aspects in 89 Patients. Rheumatology.

[8] Kahn M.E. (2003). Abstracts of the American College of Rheumatology 67th Annual Meeting and the Association of Rheumatology Health Professionals 38th Annual Meeting. October 23–28, 2003. Orlando, Florida, USA. Arthritis Rheum..

[9] Matucci-Cerinic C., Malattia C., Pistorio A., Rosina S., Consolaro A., Viola S., Volpi S., Caorsi R., Viglizzo G., Gattorno M. (2023). Skin Manifestations Help Identifying Different Phenotypes of Paediatric SAPHO Syndrome. Semin. Arthritis Rheum..

[10] Furer V., Kishimoto M., Tomita T., Elkayam O., Helliwell P.S. (2022). Pro and Contra: Is Synovitis, Acne, Pustulosis, Hyperostosis, and Osteitis (SAPHO) a Spondyloarthritis Variant?. Curr. Opin. Rheumatol..

[11] Hayem G. (2004). SAPHO syndrome. Rev. Prat..

[12] Van Doornum S., Barraclough D., McColl G., Wicks I. (2000). SAPHO: Rare or Just Not Recognized?. Semin. Arthritis Rheum..

[13] Jansson A.F., Grote V., for the ESPED Study Group (2011). Nonbacterial Osteitis in Children: Data of a German Incidence Surveillance Study. Acta Paediatr..

[14] Li Y., Li C., Wu N., Li F., Wu Z., Sun X., Li Q., Li L. (2020). Demographic, Clinical, and Scintigraphic Comparison of Patients Affected by Palmoplantar Pustulosis and Severe Acne: A Retrospective Study. Clin. Rheumatol..

[15] Sun Y., Li C., Zhu M., Zhang S., Cao Y., Yang Q., Zhao P., Huang G., Xu A. (2019). Enhanced Migration and Adhesion of Peripheral Blood Neutrophils from SAPHO Patients Revealed by RNA-Seq. Orphanet J. Rare Dis..

[16] Zhang S., Li C., Zhang S., Li L., Zhang W., Dong Z., Zhang W. (2019). Serum Levels of Proinflammatory, Anti-Inflammatory Cytokines, and RANKL/OPG in Synovitis, Acne, Pustulosis, Hyperostosis, and Osteitis (SAPHO) Syndrome. Mod. Rheumatol..

[17] Xu D., Liu X., Lu C., Luo J., Wang C., Gao C., Xie J., Li X. (2019). Reduction of Peripheral Natural Killer Cells in Patients with SAPHO Syndrome. Clin. Exp. Rheumatol..

[18] Hetrick R., Oliver M. (2023). Pediatric Autoinflammatory Bone Disorders-a Mini Review with Special Focus on Pathogenesis and Inborn Errors of Immunity. Front. Pediatr..

[19] Sharma M., Ferguson P.J. (2013). Autoinflammatory Bone Disorders: Update on Immunologic Abnormalities and Clues about Possible Triggers. Curr. Opin. Rheumatol..

[20] Garcovich S., Genovese G., Moltrasio C., Malvaso D., Marzano A.V. (2021). PASH, PAPASH, PsAPASH, and PASS: The Autoinflammatory Syndromes of Hidradenitis Suppurativa. Clin. Dermatol..

[21] Maitrepierre F., Marzano A.V., Lipsker D. (2021). A Unified Concept of Acne in the PAPA Spectrum Disorders. Dermatology.

[22] Akitsu A., Ishigame H., Kakuta S., Chung S., Ikeda S., Shimizu K., Kubo S., Liu Y., Umemura M., Matsuzaki G. (2015). IL-1 Receptor Antagonist-Deficient Mice Develop Autoimmune Arthritis Due to Intrinsic Activation of IL-17-Producing CCR2+Vγ6+γδ T Cells. Nat. Commun..

[23] Ferguson P.J., Bing X.Y., Vasef M.A., Ochoa L.A., Mahgoub A., Waldschmidt T.J., Tygrett L.T., Schlueter A.J., El-Shanti H. (2006). A Missense Mutation in *Pstpip2* Is Associated with the Murine Autoinflammatory Disorder Chronic Multifocal Osteomyelitis. Bone.

[24] Grosse J., Chitu V., Marquardt A., Hanke P., Schmittwolf C., Zeitlmann L., Schropp P., Barth B., Yu P., Paffenholz R. (2006). Mutation of Mouse Mayp/Pstpip2 Causes a Macrophage Autoinflammatory Disease. Blood.

[25] Larsen J.M. (2017). The Immune Response To *Prevotella* bacteria in Chronic Inflammatory Disease. Immunology.

[26] Hurtado-Nedelec M., Chollet-Martin S., Chapeton D., Hugot J.P., Hayem G., Gérard B. (2010). Genetic Susceptibility Factors in a Cohort of 38 Patients with SAPHO Syndrome: A Study of PSTPIP2, NOD2, and LPIN2 Genes. J. Rheumatol..

[27] Li C., Wang H., Jiang H., Shao Y., Huang G., Yuan K., Wei S. (2023). Family Aggregation and Prevalence of Other Autoimmune Diseases in SAPHO Syndrome. Heliyon.

[28] Chimenti M.S., Perricone C., D’Antonio A., Ferraioli M., Conigliaro P., Triggianese P., Ciccacci C., Borgiani P., Perricone R. (2021). Genetics, Epigenetics, and Gender Impact in Axial-Spondyloarthritis Susceptibility: An Update on Genetic Polymorphisms and Their Sex Related Associations. Front. Genet..

[29] Aljuhani F., Tournadre A., Tatar Z., Couderc M., Mathieu S., Malochet-Guinamand S., Soubrier M., Dubost J.J. (2015). The SAPHO Syndrome: A Single-Center Study of 41 Adult Patients. J. Rheumatol..

[30] Cox A.J., Darbro B.W., Laxer R.M., Velez G., Bing X., Finer A.L., Erives A., Mahajan V.B., Bassuk A.G., Ferguson P.J. (2017). Recessive Coding and Regulatory Mutations in FBLIM1 Underlie the Pathogenesis of Chronic Recurrent Multifocal Osteomyelitis (CRMO). PLoS ONE.

[31] Assmann G., Köhm M., Schuster V., Behrens F., Mössner R., Magnolo N., Oji V., Burkhardt H., Hüffmeier U. (2020). Genetic Variants in FBLIM1 Gene Do Not Contribute to SAPHO Syndrome and Chronic Recurrent Multifocal Osteomyelitis in Typical Patient Groups. BMC Med. Genet..

[32] Firinu D., Barca M.P., Lorrai M.M., Perra S., Cabras S., Muggianu E., Di Martino M.L., Manconi P.E., Del Giacco S.R. (2014). TH17 Cells Are Increased in the Peripheral Blood of Patients with SAPHO Syndrome. Autoimmunity.

[33] Rozin A.P. (2009). SAPHO Syndrome: Is a Range of Pathogen-Associated Rheumatic Diseases Extended?. Arthritis Res. Ther..

[34] Berthelot J.M., Corvec S., Hayem G. (2018). SAPHO, Autophagy, IL-1, FoxO1, and *Propionibacterium* (*Cutibacterium*) *Acnes*. Jt. Bone Spine.

[35] Trimble B.S., Evers C.J., Ballaron S.A., Young J.M. (1987). Intraarticular Injection of Propionibacterium Acnes Causes an Erosive Arthritis in Rats. Agents Actions.

[36] Perihan O., Ergul K.B., Neslihan D., Filiz A. (2012). The Activity of Adenosine Deaminase and Oxidative Stress Biomarkers in Scraping Samples of Acne Lesions. J. Cosmet. Dermatol..

[37] Moftah N.H., Hamad W.A.M., Abd Al Salam F.M., Marzouk S.A., Said M. (2011). Glutathione Peroxidase and Malondialdehyde in Skin Lesions of Acne Vulgaris. J. Egypt. Womenʼs Dermatol. Soc..

[38] Borrel V., Thomas P., Catovic C., Racine P.-J., Konto-Ghiorghi Y., Lefeuvre L., Duclairoir-Poc C., Zouboulis C.C., Feuilloley M.G.J. (2019). Acne and Stress: Impact of Catecholamines on Cutibacterium Acnes. Front. Med..

[39] Bungau A.F., Tit D.M., Stoicescu M., Moleriu L.-C., Muresan M., Radu A., Brisc M.C., Ghitea T.C. (2024). Exploring a New Pathophysiological Association in Acne Vulgaris and Metabolic Syndrome: The Role of Biogenic Amines and Glutathione Peroxidase. Medicina.

[40] Hayem G., Bouchaud-Chabot A., Benali K., Roux S., Palazzo E., Silbermann-Hoffman O., Kahn M.F., Meyer O. (1999). SAPHO Syndrome: A Long-Term Follow-up Study of 120 Cases. Semin. Arthritis Rheum..

[41] Li C., Zuo Y., Wu N., Li L., Li F., Zhang W., Xu W., Zhao X., Jing H., Pan Q. (2016). Synovitis, Acne, Pustulosis, Hyperostosis and Osteitis Syndrome: A Single Centre Study of a Cohort of 164 Patients. Rheumatology.

[42] Cao Y., Li C., Xu W., Wu X., Sun X., Zhang W., Jing H., Gu Z., Yuan S., Li L. (2019). Spinal and Sacroiliac Involvement in SAPHO Syndrome: A Single Center Study of a Cohort of 354 Patients. Semin. Arthritis Rheum..

[43] Colina M., Trotta F. (2013). Clinical and Radiological Characteristics of SAPHO Syndrome. Curr. Rheumatol. Rev..

[44] Chen X., Wang M., Cui W., Wang Z. (2021). Bone Loss in Patients with SAPHO Syndrome: A Preliminary Study. Ann. Rheum. Dis..

[45] Huang H., Zhang Z., Zhao J., Hao Y., Zhou W. (2021). The Effectiveness of Treatments for Patients with SAPHO Syndrome: A Follow-up Study of 24 Cases from a Single Center and Review of Literature. Clin. Rheumatol..

[46] Li C., Ye Y., Cao Y., Xu W., Wu N., Zhang S., Zhang W. (2020). Axial Skeletal Lesions and Disease Duration in SAPHO Syndrome: A Retrospective Review of Computed Tomography Findings in 81 Patients. Int. J. Rheum. Dis..

[47] Li C., Xu H., Gong L., Wang A., Dong X., Yuan K., Huang G., Wei S., Sun L. (2022). Work Productivity and Activity in Patients with SAPHO Syndrome: A Cross-Sectional Observational Study. Orphanet J. Rare Dis..

[48] Przepiera-Bedzak H., Brzosko M. (2021). SAPHO Syndrome: Pathogenesis, Clinical Presentation, Imaging, Comorbidities and Treatment: A Review. Postep. Dermatol. I Alergol..

[49] Sallés M., Olivé A., Perez-Andres R., Holgado S., Mateo L., Riera E., Tena X. (2011). The SAPHO Syndrome: A Clinical and Imaging Study. Clin. Rheumatol..

[50] Yap F.H.X., Olsson-White D., Roddy J., Cook N.J., Langlands D.R., Manners P.J., Carroll G.J. (2021). Long-Term Clinical Outcomes in Synovitis, Acne, Pustulosis, Hyperostosis, and Osteitis Syndrome. Mayo Clin. Proc. Innov. Qual. Outcomes.

[51] Depasquale R., Kumar N., Lalam R.K., Tins B.J., Tyrrell P.N.M., Singh J., Cassar-Pullicino V.N. (2012). SAPHO: What Radiologists Should Know. Clin. Radiol..

[52] Ferreira-Vilaca C., Costa Mendes L., Campana S.C., Bailleul-Forestier I., Audouin-Pajot C., Esclassan R., Canceill T. (2020). Orofacial Manifestations of SAPHO Syndrome: A Systematic Review of Case Reports. Clin. Rheumatol..

[53] Xu H., Chen N., Li C. (2022). Frontal Bone Involvement in SAPHO Syndrome. Clin. Rheumatol..

[54] Wang M., Li Y., Cao Y., Lu X., Liu Y., Zhao J., Zhang W., Li C. (2020). Mandibular Involvement in SAPHO Syndrome: A Retrospective Study. Orphanet J. Rare Dis..

[55] Nguyen M.T., Borchers A., Selmi C., Naguwa S.M., Cheema G., Gershwin M.E. (2012). The SAPHO Syndrome. Semin. Arthritis Rheum..

[56] Misiak-Galazka M., Wolska H., Rudnicka L. (2017). What Do We Know about Palmoplantar Pustulosis?. J. Eur. Acad. Dermatol. Venereol..

[57] Liu S., Tang M., Cao Y., Li C. (2020). Synovitis, Acne, Pustulosis, Hyperostosis, and Osteitis Syndrome: Review and Update. Ther. Adv. Musculoskelet. Dis..

[58] Leerling A.T., Navas Cañete A., Winter E.M. (2023). Chronic Nonbacterial Osteomyelitis/SAPHO-Syndrome Complicated by Subclavian Vein Obstruction. Rheumatology.

[59] Sanges S., Ducoulombier V., Sivery B., Delhaye N., Haffner C., Houvenagel E. (2015). Thrombosis of the Subclavian Vein Complicating a SAPHO Syndrome: An Observation. Rev. Rhum..

[60] Shiraishi W., Hayashi S., Iwanaga Y., Murai H., Yamamoto A., Kira J. (2015). A Case of Synovitis, Acne, Pustulosis, Hyperostosis, and Osteitis (SAPHO) Syndrome Presenting with Hypertrophic Pachymeningitis. J. Neurol. Sci..

[61] Baisya R., Gavali M., Tyagi M., Devarasetti P.K. (2023). A Case of SAPHO Syndrome Complicated by Uveitis with Good Response to Both TNF Inhibitor and JAKinib. Case Rep. Rheumatol..

[62] Correia C.P., Martins A., Oliveira J., Andrade S., Almeida J. (2019). Systemic Amyloidosis with Renal Failure: A Challenging Diagnosis of SAPHO Syndrome. Eur. J. Case Rep. Intern. Med..

[63] Hasegawa S., Yabe H., Kaneko N., Watanabe E., Gono T., Terai C. (2017). Synovitis-Acne-Pustulosis-Hyperostosis-Osteitis (SAPHO) Syndrome with Significant Bilateral Pleural Effusions. Intern. Med..

[64] Li C., Liu S., Sui X., Wang J., Song W., Xu W., Xu K.-F., Tian X., Zhang W. (2018). Pulmonary High-Resolution Computed Tomography Findings in Patients with Synovitis, Acne, Pustulosis, Hyperostosis and Osteitis Syndrome. PLoS ONE.

[65] Demirci Yildirim T., Sari I. (2024). SAPHO Syndrome: Current Clinical, Diagnostic and Treatment Approaches. Rheumatol. Int..

[66] Earwaker J.W.S., Cotten A. (2003). SAPHO: Syndrome or Concept? Imaging Findings. Skelet. Radiol..

[67] Quirico Rodríguez M., Casáns Tormo I., Redal Peña M.C., López Castillo V. (2010). The importance of bone scintigraphy in the diagnosis of SAPHO syndrome. Rev. Esp. Med. Nucl..

[68] Fritz J. (2015). The Contributions of Whole-Body Magnetic Resonance Imaging for the Diagnosis and Management of Chronic Recurrent Multifocal Osteomyelitis. J. Rheumatol..

[69] Fritz J., Tzaribatchev N., Claussen C.D., Carrino J.A., Horger M.S. (2009). Chronic Recurrent Multifocal Osteomyelitis: Comparison of Whole-Body MR Imaging with Radiography and Correlation with Clinical and Laboratory Data. Radiology.

[70] Zhang L.H., Han S.B., Song L., Gao S., Zhao Q., Deng X.L., Yuan H.S. (2021). Comparative Analysis and Differentiation between SAPHO Syndrome and Spondyloarthropathies Using Whole-Spine MRI. Clin. Radiol..

[71] Patel C.N., Smith J.T., Rankine J.J., Scarsbrook A.F. (2009). F-18 FDG PET/CT Can Help Differentiate SAPHO Syndrome from Suspected Metastatic Bone Disease. Clin. Nucl. Med..

[72] Umeda M., Kawashiri S.Y., Nishino A., Koga T., Ichinose K., Michitsuji T., Shimizu T., Fukui S., Nakashima Y., Hirai Y. (2017). Synovitis of Sternoclavicular and Peripheral Joints Can Be Detected by Ultrasound in Patients with SAPHO Syndrome. Mod. Rheumatol..

[73] Przepiera-Będzak H., Brzosko M. (2018). Clinical Symptoms, Imaging, and Treatment of SAPHO Syndrome: A Single-center Study of 52 Cases. Pol. Arch. Intern. Med..

[74] Valkema P.A., Luymes C.H., Witteveen J.E., le Cessie S., Appelman-Dijkstra N.M., Hogendoorn P.C., Hamdy N.A. (2017). High Prevalence of Autoimmune Disease in the Rare Inflammatory Bone Disorder Sternocostoclavicular Hyperostosis: Survey of a Dutch Cohort. Orphanet J. Rare Dis..

[75] Li C., Jiang H., Zhang Y., Huang G. (2023). Sexual Function Assessment in Patients with SAPHO Syndrome: A Cross-Sectional Study. Orphanet J. Rare Dis..

[76] Xiang Y., Jiao R., Cao Y., Liang D., Zhang W., Yu Y., Zhang W., Li C. (2021). Fibromyalgia in Patients with Synovitis, Acne, Pustulosis, Hyperostosis, and Osteitis (SAPHO) Syndrome: Prevalence and Screening. Clin. Rheumatol..

[77] Kahn M.F., Khan M.A. (1994). The SAPHO Syndrome. Baillieres Clin. Rheumatol..

[78] Przepiera-Będzak H., Fischer K., Brzosko M. (2021). Serum Interleukin-23 Protects, Whereas Methotrexate Treatment Stimulates Selected Components of the Metabolic Syndrome in Patients with SAPHO Syndrome. Arch. Med. Sci..

[79] Resorlu H., Inceer B.S., Kılıc S., Isık S. (2017). Pustulotic Arthro-Osteitis (Sonozaki Syndrome): A Rare Case Report. J. Back. Musculoskelet. Rehabil..

[80] Yamamoto T. (2013). Pustulotic Arthro-Osteitis Associated with Palmoplantar Pustulosis. J. Dermatol..

[81] Yamamoto T. (2019). Clinical Characteristics of Japanese Patients with Palmoplantar Pustulosis. Clin. Drug Investig..

[82] Colina M., Trotta F. (2014). Antibiotics May Be Useful in the Treatment of SAPHO Syndrome. Mod. Rheumatol..

[83] Andreasen C.M., Jurik A.G., Deleuran B.W., Horn H.C., Folkmar T.B., Herlin T., Hauge E.M. (2020). Pamidronate in Chronic Non-Bacterial Osteomyelitis: A Randomized, Double-Blinded, Placebo-Controlled Pilot Trial. Scand. J. Rheumatol..

[84] Amital H., Applbaum Y.H., Aamar S., Daniel N., Rubinow A. (2004). SAPHO Syndrome Treated with Pamidronate: An Open-Label Study of 10 Patients. Rheumatology.

[85] Bhat C.S., Roderick M., Sen E.S., Finn A., Ramanan A.V. (2019). Efficacy of Pamidronate in Children with Chronic Non-Bacterial Osteitis Using Whole Body MRI as a Marker of Disease Activity. Pediatr. Rheumatol. Online J..

[86] Colina M., La Corte R., Trotta F. (2009). Sustained Remission of SAPHO Syndrome with Pamidronate: A Follow-up of Fourteen Cases Axnd a Review of the Literature. Clin. Exp. Rheumatol..

[87] Delattre E., Guillot X., Godfrin-Valnet M., Prati C., Wendling D. (2015). SAPHO Syndrome Treatment with Intravenous Pamidronate. Retrospective Study of 22 Cases. Rev. Rhum..

[88] Sakellariou G.T., Chaitidis N., Tsifountoudis I. (2023). Follow-up Imaging of SAPHO Syndrome Treated with Denosumab. Rheumatology.

[89] Azevedo V.F., Dal Pizzol V.I., Lopes H., Coelho S.P., Czeczko L.E. (2011). Methotrexate to treat SAPHO syndrome with keloidal scars. Acta Reum. Port..

[90] Grosjean C., Hurtado-Nedelec M., Nicaise-Roland P., Ferreyra-Dillon R., Bollet C., Quintin E., Dieude P., Palazzo E., Wattiaux M.J., Kahn M.F. (2010). Prevalence of Autoantibodies in SAPHO Syndrome: A Single-Center Study of 90 Patients. J. Rheumatol..

[91] Vaccaro M., Borgia F., Guarneri F., Blandino A., Cannavò S.P., Guarneri B. (2001). Successful Treatment of Pustulotic Arthro-Osteitis (Sonozaki Syndrome) with Systemic Cyclosporin. Clin. Exp. Dermatol..

[92] Scarpato S., Tirri E. (2005). Successful Treatment of SAPHO Syndrome with Leflunomide. Report of Two Cases. Clin. Exp. Rheumatol..

[93] Li Z., Liu S., Liu Y., Ma M., Li L., Li C. (2023). Successful Treatment of Nail Involvement Using Leflunomide in a Patient with Synovitis, Acne, Pustulosis, Hyperostosis and Osteitis (SAPHO) Syndrome. Australas. J. Dermatol..

[94] Adamo S., Nilsson J., Krebs A., Steiner U., Cozzio A., French L.E., Kolios A.G.A. (2018). Successful Treatment of SAPHO Syndrome with Apremilast. Br. J. Dermatol..

[95] Zhang J., Li Y., Wang J., Jiang H., Qi J., Li C., Ying Z. (2024). Is Apremilast a Treatment for SAPHO Syndrome?. J. Dermatol. Treat..

[96] Ben Abdelghani K., Dran D.G., Gottenberg J.-E., Morel J., Sibilia J., Combe B. (2010). Tumor Necrosis Factor-α Blockers in SAPHO Syndrome: Table 1. J. Rheumatol..

[97] Cianci F., Zoli A., Gremese E., Ferraccioli G. (2017). Clinical Heterogeneity of SAPHO Syndrome: Challenging Diagnose and Treatment. Clin. Rheumatol..

[98] Henriques C.C., Sousa M., Panarra A., Riso N. (2011). The Dark Side of SAPHO Syndrome. Case Rep..

[99] Cotti E., Careddu R., Schirru E., Marongiu S., Barca M.P., Manconi P.E., Mercuro G. (2015). A Case of SAPHO Syndrome with Endodontic Implications and Treatment with Biologic Drugs. J. Endod..

[100] Castellví I., Bonet M., Narváez J.A., Molina-Hinojosa J.C. (2010). Successful Treatment of SAPHO Syndrome with Adalimumab: A Case Report. Clin. Rheumatol..

[101] Arias-Santiago S., Sanchez-Cano D., Callejas-Rubio J., Fernández-Pugnaire M., Ortego-Centeno N. (2010). Adalimumab Treatment for SAPHO Syndrome. Acta Derm. Venerol..

[102] Garcovich S., Amelia R., Magarelli N., Valenza V., Amerio P. (2012). Long-Term Treatment of Severe SAPHO Syndrome with Adalimumab: Case Report and a Review of the Literature. Am. J. Clin. Dermatol..

[103] Vekic D.A., Woods J., Lin P., Cains G.D. (2018). SAPHO Syndrome Associated with Hidradenitis Suppurativa and Pyoderma Gangrenosum Successfully Treated with Adalimumab and Methotrexate: A Case Report and Review of the Literature. Int. J. Dermatol..

[104] Yang Q., Xiang T., Wu Y., Ye E., He B., Bu Z. (2022). SAPHO Syndrome with Palmoplantar Pustulosis as the First Manifestation Successfully Treated with Adalimumab. Clin. Cosmet. Investig. Dermatol..

[105] Maccora I., Marrani E., Maniscalco V., Mastrolia M.V., Pagnini I., Simonini G. (2021). Diagnostic Challenge of Synovitis, Acne, Pustulosis, Hyperostosis, and Osteitis (SAPHO) Syndrome in Pediatric Age: A Monocentric Case Series. Modern Rheumatology.

[106] Kanda R., Nakano K., Miyagawa I., Iwata S., Nakayamada S., Tanaka Y. (2020). A Case of Bone Destruction Caused by Chronic Non-Bacterial Osteomyelitis (CNO) Successfully Repaired with a Tumour Necrosis Factor-α (TNF-α) Inhibitor, Adalimumab. Mod. Rheumatol. Case Rep..

[107] Genovese G., Caorsi R., Moltrasio C., Marzano A.V. (2019). Successful Treatment of Co-existent SAPHO Syndrome and Hidradenitis Suppurativa with Adalimumab and Methotrexate. Acad. Dermatol. Venereol..

[108] Crowley E.L., O’Toole A., Gooderham M.J. (2018). Hidradenitis Suppurativa with SAPHO Syndrome Maintained Effectively with Adalimumab, Methotrexate, and Intralesional Corticosteroid Injections. SAGE Open Med. Case Rep..

[109] Ferraioli M., Fiannacca L., Greco E., Cela E., Fatica M., Bergamini A., Chimenti M.S. (2024). Risankizumab Efficacy in Synovitis, Acne, Pustulosis, Hyperostosis, and Osteitis (SAPHO) Remission: A Case Report on Rheumatologic and Dermatologic Disease Manifestations with Literature Review. Case Rep. Immunol..

[110] Fan D., Li F., Liu Z., Tang Z., Lv S. (2024). Successful Treatment of Refractory Synovitis, Acne, Pustulosis, Hyperostosis, and Osteitis (SAPHO) Syndrome and Paradoxical Psoriasis with Secukinumab: A Case Report. Clin. Cosmet. Investig. Dermatol..

[111] Sun B., Cao Y., Wang L., Wang M., Li C. (2021). Successful Treatment of Refractory Mandibular Lesions in SAPHO Syndrome with Secukinumab. Rheumatology.

[112] Figueiredo A.S.B., Oliveira A.L., Caetano A., Moraes-Fontes M.F. (2020). SAPHO: Has the Time Come for Tailored Therapy?. Clin. Rheumatol..

[113] Marrani E., Belli G., Simonini G., Trapani S., Caproni M., Lionetti P. (2018). SAPHO Syndrome in Pediatric Patients with Inflammatory Bowel Disease Treated with Infliximab. Dig. Liver Dis..

[114] Massara A., Cavazzini P.L., Trotta F. (2006). In SAPHO Syndrome Anti-TNF-α Therapy May Induce Persistent Amelioration of Osteoarticular Complaints, but May Exacerbate Cutaneous Manifestations. Rheumatology.

[115] Burgemeister L.T., Baeten D.L., Tas S.W. (2012). Biologics for rare inflammatory diseases: TNF blockade in the SA PHO syndrome. Neth. J. Med..

[116] Gupta A.K., Skinner A.R. (2004). A Review of the Use of Infliximab to Manage Cutaneous Dermatoses. J. Cutan. Med. Surg..

[117] Olivieri I. (2002). Successful Treatment of SAPHO Syndrome with Infliximab: Report of Two Cases. Ann. Rheum. Dis..

[118] Moll C., Hernández M.V., Cañete J.D., Gómez-Puerta J.A., Soriano A., Collado A., Sanmartí R. (2008). Ilium Osteitis as the Main Manifestation of the SAPHO Syndrome: Response to Infliximab Therapy and Review of the Literature. Semin. Arthritis Rheum..

[119] Anić B., Padjen I., Barešić M., Težak S. (2014). The Lobster Sign in SAPHO Syndrome: Unusually Extensive Osteitis of the Anterior Chest Wall Partially Responsive to Infliximab. Rheumatol. Int..

[120] De Souza A., Solomon G.E., Strober B.E. (2011). SAPHO Syndrome Associated with Hidradenitis Suppurativa Successfully Treated with Infliximab and Methotrexate. Bull. NYU Hosp. Jt. Dis..

[121] Fruehauf J., Cierny-Modre B., Caelen L.E.-S., Schwarz T., Weinke R., Aberer E. (2009). Response to Infliximab in SAPHO Syndrome. Case Rep..

[122] Iqbal M., Kolodney M.S. (2005). Acne Fulminans with Synovitis-Acne-Pustulosis-Hyperostosis-Osteitis (SAPHO) Syndrome Treated with Infliximab. J. Am. Acad. Dermatol..

[123] Sabugo F., Liberman C., Niedmann J.P., Soto L., Cuchacovich M. (2008). Infliximab Can Induce a Prolonged Clinical Remission and a Decrease in Thyroid Hormonal Requirements in a Patient with SAPHO Syndrome and Hypothyroidism. Clin. Rheumatol..

[124] Mateo L., Sanint J., Rodríguez Muguruza S., Martínez Morillo M., Pérez Andrés R., Domenech Puigcerver S. (2017). Lesión osteolítica cervical como presentación del síndrome SAPHO. Reumatol. Clínica.

[125] Hampton S.L., Youssef H. (2013). Successful Treatment of Resistant SAPHO Syndrome with Anti-TNF Therapy. BMJ Case Rep..

[126] Wagner A.D., Andresen J., Jendro M.C., Hülsemann J.L., Zeidler H. (2002). Sustained Response to Tumor Necrosis Factor α–Blocking Agents in Two Patients with SAPHO Syndrome. Arthritis Rheum..

[127] Ito H., Hirano Y. (2024). Long-Term Clinical Course of Two Rare Cases of Synovitis-Acne-Pustulosis-Hyperostosis-Osteomyelitis Syndrome Involving Only Unilateral Femur. Mod. Rheumatol. Case Rep..

[128] Deutschmann A., Mache C.J., Bodo K., Zebedin D., Ring E. (2005). Successful Treatment of Chronic Recurrent Multifocal Osteomyelitis With Tumor Necrosis Factor-α Blockage. Pediatrics.

[129] Abourazzak F.E., Hachimi H., Kadi N., Berrada K., Tizniti S., Harzy T. (2014). Etanercept in the Treatment of SAPHO Syndrome: Which Place?. Eur. J. Rheumatol..

[130] Zhang L.L., Zhao J.X., Liu X.Y. (2012). Successful Treatment of SAPHO Syndrome with Severe Spinal Disorder Using Entercept: A Case Study. Rheumatol. Int..

[131] Zhang L., Gao Z. (2016). Etanercept in the Treatment of Refractory SAPHO Syndrome. Am. J. Clin. Exp. Immunol..

[132] Vilar-Alejo J., Dehesa L., De La Rosa-del Rey P., Novoa-Medina J., Valerón-Almazán P., Santana Medina N., Bastida J. (2010). SAPHO Syndrome with Unusual Cutaneous Manifestations Treated Successfully with Etanercept. Acta Derm. Venerol..

[133] Su Y.-S., Chang C.-H. (2015). SAPHO Syndrome Associated with Acne Conglobata Successfully Treated with Etanercept. J. Formos. Med. Assoc..

[134] Marí A., Morla A., Melero M., Schiavone R., Rodríguez J. (2014). Diffuse Sclerosing Osteomyelitis (DSO) of the Mandible in SAPHO Syndrome: A Novel Approach with Anti-TNF Therapy. Systematic Review. J. Cranio-Maxillofac. Surg..

[135] Sáez-Martín L., Gómez-Castro S., Román-Curto C., Palacios-Álvarez I., Fernández-López E. (2015). Etanercept in the Treatment of SAPHO Syndrome. Int. J. Dermatol..

[136] Li C., Wu X., Cao Y., Zeng Y., Zhang W., Zhang S., Liu Y., Jin H., Zhang W., Li L. (2019). Paradoxical Skin Lesions Induced by Anti-TNF-α Agents in SAPHO Syndrome. Clin. Rheumatol..

[137] Gimeno-Castillo J., Rosés-Gibert P., Parrón A.M., de la Torre Gomar F.J., de Lagrán-Álvarez de Arcaya Z.M. (2022). Paradoxical SAPHO Syndrome after Etanercept in a Patient with Psoriasis. Indian. J. Dermatol..

[138] Wang G., Zhuo N., Li J.Y. (2022). Off-Label Use of Secukinumab: A Potential Therapeutic Option for SAPHO Syndrome. J. Rheumatol..

[139] Wendling D., Aubin F., Verhoeven F., Prati C. (2018). Targeting the IL-23/Th17 axis in the treatment of SAPHO syndrome. Rev. Du Rhum..

[140] Wang L., Sun B., Li C. (2021). Clinical and Radiological Remission of Osteoarticular and Cutaneous Lesions in SAPHO Patients Treated With Secukinumab: A Case Series. J. Rheumatol..

[141] Tu W., Nie D., Chen Y., Wen C., Zeng Z. (2023). Successful Treatment of SAPHO Syndrome Complicated with Ankylosing Spondylitis by Secukinumab: A Case Report. J. Pers. Med..

[142] Ji Q., Wang Q., Pan W., Hou Y., Wang X., Bian L., Wang Z. (2022). Exceptional Response of Skin Symptoms to Secukinumab Treatment in a Patient with SAPHO Syndrome: Case Report and Literature Review. Medicine.

[143] Nikolakis G., Kreibich K., Vaiopoulos A., Kaleta K., Talas J., Becker M., Zouboulis C.C. (2021). Case Report: PsAPSASH Syndrome: An Alternative Phenotype of Syndromic Hidradenitis Suppurativa Treated with the IL-17A Inhibitor Secukinumab. F1000Research.

[144] Xia R., Diao Z., Zhang Z., Liu J., Yin Z. (2022). Successful Treatment of Synovitis, Acne, Pustulosis, Hyperostosis and Osteitis Syndrome with Ixekizumab. Clin. Exp. Dermatol..

[145] Passeron T., Perrot J.-L., Jullien D., Goujon C., Ruer M., Boyé T., Villani A.P., Quiles Tsimaratos N. (2024). Treatment of Severe Palmoplantar Pustular Psoriasis with Bimekizumab. JAMA Dermatol..

[146] D’Ignazio E., Trovato E., Cantarini L., Calabrese L., Frediani B., Gentileschi S. (2024). Clinical response to brodalumab in a patient affected by refractory SAPHO syndrome. Clin. Exp. Rheumatol..

[147] Funabiki M., Tahara M., Kondo S., Ayuzawa N., Yanagida H. (2023). SAPHO Syndrome Complicated by Lesions of the Central Nervous System Successfully Treated with Brodalumab. Case Rep. Rheumatol..

[148] Flora A., Holland R., Smith A., Frew J.W. (2021). Rapid and sustained remission of synovitis, acne, pustulosis, hyperostosis, and osteitis (SAPHO) syndrome with IL-23p19 antagonist (risankizumab). JAAD Case Rep..

[149] Wendling D., Prati C., Aubin F. (2012). Anakinra Treatment of SAPHO Syndrome: Short-Term Results of an Open Study. Ann. Rheum. Dis..

[150] Yue Q., Ma M., Liu S., Wu Y., Li C. (2023). JAKi: Can It Be Used to Treat SAPHO Syndrome?. Int. J. Rheum. Dis..

[151] Li Y., Huo J., Cao Y., Yu M., Zhang Y., Li Z., Li C., Zhang W. (2020). Efficacy of Tofacitinib in Synovitis, Acne, Pustulosis, Hyperostosis and Osteitis Syndrome: A Pilot Study with Clinical and MRI Evaluation. Ann. Rheum. Dis..

[152] Li C., Li Z., Cao Y., Li L., Li F., Li Y., Xiong D., Wu X., Zhang W., Zeng X. (2021). Tofacitinib for the Treatment of Nail Lesions and Palmoplantar Pustulosis in Synovitis, Acne, Pustulosis, Hyperostosis, and Osteitis Syndrome. JAMA Dermatol..

[153] Ru C., Qian T., Liu X., Wang C., Li W., Hou X., Li C. (2023). SAPHO Syndrome with Takayasu Arteritis Successfully Treated with Tofacitinib. Int. J. Rheum. Dis..

[154] Dierckx S., Nisolle J.-F., Boutsen Y. (2024). Dramatic Response of Synovitis, Acne, Pustulosis, Hyperostosis, and Osteitis Syndrome to Tofacitinib Monotherapy: A Case Report. J. Med. Case Rep..

[155] Yang Q., Zhao Y., Li C., Luo Y., Hao W., Zhang W. (2018). Case Report: Successful Treatment of Refractory SAPHO Syndrome with the JAK Inhibitor Tofacitinib. Medicine.

[156] Wu X., Cao Y., Li C. (2022). Successful treatment of hip involvement in SAPHO syndrome with baricitinib. Rheumatol. Adv. Pr..

[157] Liu S., Yu Y., Liu Y., Ma M., Li C. (2023). Efficacy of Baricitinib in Synovitis, Acne, Pustulosis, Hyperostosis, and Osteitis Syndrome: A Case Series. Jt. Bone Spine.

[158] Ma M., Lu S., Hou X., Li C. (2023). Novel JAK-1 Inhibitor Upadacitinib as a Possible Treatment for Refractory SAPHO Syndrome: A Case Report. Int. J. Rheum. Dis..

[159] Wang L., Gong L., Zhang X., Cao Y., Long P., Zhang W., Zeng X., Li C. (2021). Tripterygium Wilfordii Hook F. in the Treatment of Synovitis, Acne, Pustulosis, Hyperostosis, and Osteitis Syndrome: A Clinical Trial. Clin. Rheumatol..

[160] Zhang X., Wu X., Li C. (2020). Successful Treatment of Synovitis, Acne, Pustulosis, Hyperostosis, and Osteitis and Paradoxical Skin Lesions by Tripterygium Wilfordii Hook f: A Case Report. J. Int. Med. Res..

[161] Radu A., Tit D.M., Endres L.M., Radu A.-F., Vesa C.M., Bungau S.G. (2024). Naturally Derived Bioactive Compounds as Precision Modulators of Immune and Inflammatory Mechanisms in Psoriatic Conditions. Inflammopharmacol.

[162] Behl T., Mehta K., Sehgal A., Singh S., Sharma N., Ahmadi A., Arora S., Bungau S. (2022). Exploring the Role of Polyphenols in Rheumatoid Arthritis. Crit. Rev. Food Sci. Nutr..

[163] Zhang S., Wang J., Liu L., Sun X., Zhou Y., Chen S., Lu Y., Cai X., Hu M., Yan G. (2022). Efficacy and Safety of Curcumin in Psoriasis: Preclinical and Clinical Evidence and Possible Mechanisms. Front. Pharmacol..

[164] Kawakami H., Nagaoka Y., Hirano H., Matsumoto Y., Abe N., Tsuboi R., Kanno Y., Okubo Y. (2019). Evaluation of the Efficacy of Granulocyte and Monocyte Adsorption Apheresis on Skin Manifestation and Joint Symptoms of Patients with Pustulotic Arthro-Osteitis. J. Dermatol..

[165] Reynolds R.V., Yeung H., Cheng C.E., Cook-Bolden F., Desai S.R., Druby K.M., Freeman E.E., Keri J.E., Stein Gold L.F., Tan J.K.L. (2024). Guidelines of Care for the Management of Acne Vulgaris. J. Am. Acad. Dermatol..

[166] Divya B.L., Rao P.N. (2016). SAPHO Syndrome with Acne Fulminans and Severe Polyosteitis Involving Axial Skeleton. Indian. Dermatol. Online J..

[167] Freira S., Fonseca H., Ferreira P.D., Vasconcelos P., Fonseca J.E. (2014). SAPHO Syndrome in an Adolescent: A Clinical Case with Unusual Severe Systemic Impact. J. Adolesc. Health.

[168] Galadari H., Bishop A.G., Venna S.S., Sultan E., Do D., Zeltser R. (2009). Synovitis, Acne, Pustulosis, Hyperostosis, and Osteitis Syndrome Treated with a Combination of Isotretinoin and Pamidronate. J. Am. Acad. Dermatol..

[169] Greywal T., Zaenglein A.L., Baldwin H.E., Bhatia N., Chernoff K.A., Del Rosso J.Q., Eichenfield L.F., Levin M.H., Leyden J.J., Thiboutot D.M. (2017). Evidence-Based Recommendations for the Management of Acne Fulminans and Its Variants. J. Am. Acad. Dermatol..

[170] Karatas Togral A., Yıldızgoren M.T., Koryurek O.M., Ekiz T. (2015). Can Isotretinoin Induce Articular Symptoms in SAPHO Syndrome?. West. Indian. Med. J..

[171] Luzzati M., Simonini G., Filippeschi C., Giani T., Trapani S. (2020). SAPHO Syndrome: The Supposed Trigger by Isotretinoin, the Efficacy of Adalimumab and the Specter of Depressive Disorder: A Case Report. Ital. J. Pediatr..

[172] Shahada O.O., Kurdi A.S., Aljawi A.F., Khayat L.I., Shahadah A.O. (2022). Synovitis, Acne, Pustulosis, Hyperostosis, and Osteitis Syndrome Diagnosis in Adolescent and Isotretinoin as a Possible Serious Exacerbating Factor. Cureus.

[173] Xiang Y., Wang Y., Cao Y., Li Z., Xiong D., Wang L., Zhang W., Zeng X., Wang Y., Li C. (2021). Tonsillitis as a Possible Predisposition to Synovitis, Acne, Pustulosis, Hyperostosis and Osteitis (SAPHO) Syndrome. Int. J. Rheum. Dis..

[174] Horiguchi S., Fujita T., Kinoshita K., Doi K. (2020). Tonsillectomy as an Effective Treatment for Arthralgia of SAPHO Syndrome. J. Surg. Case Rep..

[175] Witt M., Meier J., Hammitzsch A., Proft F., Schulze-Koops H., Grunke M. (2014). Disease Burden, Disease Manifestations and Current Treatment Regimen of the SAPHO Syndrome in Germany: Results from a Nationwide Patient Survey. Semin. Arthritis Rheum..

[176] Ramautar A.I., Appelman-Dijkstra N.M., Lakerveld S., Schroijen M.A., Snel M., Winter E.M., Hamdy N.A. (2021). Chronic Nonbacterial Osteomyelitis of the Sternocostoclavicular Region in Adults: A Single-Center Dutch Cohort Study. JBMR Plus.

