# Supplementary material for: What Is New and What Is Next for SAPHO Syndrome Management: A Narrative Review
Source: J Clin Med. 2025 Feb 18;14(4):1366. doi: 10.3390/jcm14041366 (PMC11856149; doi:10.3390/jcm14041366)
Supplement: Supplementary file 1 [file jcm-14-01366-s001.zip › jcm-3407778-supplementary.pdf]

Supplementary Table S1: Reports on biologic and small molecules drugs used in SAPHO syndrome.

Author	Article Type	Number of treated patients	Efficacy on skin symptoms	Efficacy on osteoarticular symptoms
**Anti-TNF**				
**Adalimumab**				
Ben Abdelghani *et al.* (2010) [96]	CR	2	1/1	1/2
Cianci *et al.* (2017) [97]	CR	1	-	1/1
Henriques *et al.* (2011) [98]	CR	1	0/1	0/1
Cotti *et al.* (2015) [99]	CR	1^	-	1/1
Castellví *et al.* (2010) [100]	CR	1	1/1	1/1
Arias-Santiago *et al.* (2010) [101]	CR	1	1/1	1/1
Garcovich *et al.* (2012) [102]	CR	1	1/1	1/1
Vekic *et al.* (2018) [103]	CR	1^	1/1	1/1
Matucci-Cerinic *et al.* (2023) [9]	CS	8^	6/8	8/8
Yang *et al.* (2022) [104]	CR	1	1/1	1/1
Maccora *et al.* (2021) [105]	CS	4	4/4	4/4
Kanda *et al.* (2020) [106]	CR	1	1/1	1/1
Genovese *et al.* (2019) [107]	CR	1	1/1	1/1
Crowley *et al.* (2018) [108]	CR	1^	1/1	1/1
Ferraioli *et al.* (2024) [109]	CR	1	1 (partial)/1	1 (partial)/1
Fan *et al.* (2024) [110]	CR	1	1 (partial)/1	1/1
Sun *et al.* (2021) [111]	CR	1	-	0/1
Figueiredo *et al.* (2020) [112]	CR	1	1/1	1/1
Marrani *et al.* (2018) [113]	CR	1	1/1	1/1
Huang *et al.* (2021) [45]	CS	1^*	1/1	1/1
**Tot**		31	24/27	28/31
**Infliximab**				
Massara *et al.* (2006) [114]	CS	4	1/3	4/4
Ben Abdelghani *et al.* (2010) [96]	CS	4	1/4	1/3
Burgemeister *et al.* (2012) [115]	CS	3	2/2	3/3
Gupta *et al.* (2004) [116]	CS	3	1/1	3/3
Olivieri *et al.* (2002) [117]	CS	2	1/1	2/2
Moll *et al.* (2008) [118]	CS	2	1/1	2/2
Anić *et al.* (2014) [119]	CR	1	1/1	1/1
De Souza *et al.* (2011) [120]	CR	1	1/1	1/1
Fruehauf *et al.* (2009) [121]	CR	1	1/1	1 (partial)/1
Iqbal *et al.* (2005) [122]	CR	1^	1/1	1/1
Sabugo *et al.* (2008) [123]	CR	1	1/1	1/1
Mateo *et al.* (2017) [124]	CR	1	0/1	1/1
Hampton *et al.* (2013) [125]	CR	1	-	1/1
Wagner *et al.* (2002) [126]	CR	1	-	1/1
Ito *et al.* (2024) [127]	CR	1	1/1	1/1
Deutschman *et al.* (2005) [128]	CR	1	-	1/1
Arias-Santiago *et al.* (2010) [101]	CR	1	1/1	0/1
**Tot**		28	14/20	25/28
**Etanercept**				
Ben Abdelghani *et al* (2010) [96]	CS	3	2/3	2/2
Wagner *et al.* (2002) [126]	CR	1	-	1/1
Abouzzarrak *et al.* (2014) [129]	CR	1	1/1	1/1
Matucci-Cerinic *et al.* (2023) [9]	CS	5	0/4	5/5
Maccora *et al.* (2021) [105]	CS	1	1/1	1/1
Huang *et al.* (2021) [45]	CS	3^*	3/3	3/3
Zhang *et al.* (2012) [130]	CR	1*	1/1	1/1
Zhang *et al.* (2016) [131]	CS	2	2/2	2/2
Vilar-Alejo *et al.* (2010) [132]	CR	1	1/1	1/1
Su *et al.* (2015) [133]	CR	1	1/1	1/1
Marí *et al.* (2014) [134]	CR	1	-	1/1
Sàez-Martin *et al.* (2015) [135]	CR	1	1/1	1/1
Figueiredo *et al.* (2020) [112]	CR	1	0/1	1/1
**Tot**		22	13/19	21/21
**Anti-IL-17**				
**Secukinumab**				
Wendling *et al.* (2018) [139]	CS	3	2/3	0/3
Wang *et al.* (2021) [138]	CS	4	4/4	4/4

Ferraioli *et al.* (2024) [109]	CR	1	0/1	0/1
Fan *et al.* (2024) [110]	CR	1	1/1	1/1
Tu *et al.* (2023) [141]	CR	1	1/1	1/1
Ji *et al.* (2022) [142]	CR	1	1/1	1/1
Nikolakis *et al.* (2021) [143]	CR	1	1/1	1/1
Sun *et al.* (2021) [111]	CR	1	-	1/1
Matucci-Cerinic *et al.* (2023) [9]	CS	2	1/2	1/2
**Tot**		15	11/14	10/15
**Ixekizumab**				
Xia *et al.* (2022) [144]	CR	1	1/1	1/1
**Brodalumab**				
D'Ignazio *et al.* (2024) [146]	CR	1	1/1	1/1
Funabiki *et al.* (2023) [147]	CR	1	-	1/1
**Anti-IL-12/23p40**				
**Ustekinumab**				
Wendling *et al.* (2018) [129]	CS	3	1/3	1/3
Figueiredo *et al.* (2020) [112]	CR	1^	1/1	1/1
Matucci-Cerinic *et al.* (2023) [9]	CS	2	1 (partial)/2	0/2
**Tot**		6	3/6	2/6
**Risankizumab**				
Ferraioli *et al.* (2024) [109]	CR	1	1/1	1/1
Flora *et al.* (2021) [148]	CR	1	1/1	1/1
**anti-IL-1**				
**Anakinra**				
Wendling *et al.* (2012) [149]	CS	6	5/6	5/6
**JAK inhibitors**				
**Tofacitinib**				
Li *et al.* (2020) [151]	Pilot study	12	7/8	9/12
Li *et al.* (2021) [152]	Pilot study	13	13/13	-
Dierckx *et al.* (2024) [154]	CR	1	-	1/1
Matucci-Cerinic *et al.* (2023) [9]	CS	1	0/1	0/1
Yang *et al.* (2018) [155]	CR	1	-	1/1
Ru *et al.* (2023) [153]	CR	1	1/1	1/1
**Tot**		29	21/23	12/16
**Upatacitinib**				
Ma *et al.* (2023) [158]	CR	1	1/1	1/1
**Baricitinib**				
Liu *et al.* (2023) [157]	CS	5	5/5	5/5
Wu *et al.* (2022) [156]	CS	1	0/1	1/1

Table's Legend: CR: case report; CS: case series; *with other drugs (sulfasalazine, Cyclosporin A), ^ with Methotrexate
